# Case report: Diagnosis of hereditary hemorrhagic telangiectasia (Osler Weber Rendu Syndrome) in a 23-year-old male presented with anemia and thrombocytopenia and its response to bevacizumab

**DOI:** 10.3389/fmed.2022.1001695

**Published:** 2022-10-03

**Authors:** Hamza Yunus, Said Amin, Furqan Ul Haq, Waqar Ali, Tanveer Hamid, Wajid Ali, Basharat Ullah, Payal Bai

**Affiliations:** ^1^Internal Medicine, Hayatabad Medical Complex, Peshawar, KPK, Pakistan; ^2^Internal Medicine, Mardan Medical Complex, Mardan, KPK, Pakistan; ^3^Internal Medicine, Sligo General Hospital, Sligo, Ireland; ^4^Pediatric Oncology, Shaukat Khanum Memorial Hospital and Research Centre, Peshawar, KPK, Pakistan; ^5^Peoples University of Medical and Health Sciences (PUMHS), Nawabshah, Pakistan

**Keywords:** hereditary hemorrhagic telangiectasia, Osler Weber Rendu Syndrome, diagnosis, management, bevacizumab, case report, gastrointestinal bleeding, refractory anemia

## Abstract

Osler Weber Rendu Syndrome (OWS) is characterized by the development of abnormally dilated blood vessels, which manifest as arteriovenous shunts (pulmonary, gastrointestinal, hepatic, and cerebral) and mucocutaneous telangiectasias (lips, tongue, and fingertips). It is an autosomal dominant disease with a defect in transforming growth factor beta superfamily genes. This defect results in increased angiogenesis and disruption of vessel wall integrity. The disease remains underreported, with occasional history of recurrent epistaxis, iron deficiency anemia, and gastrointestinal bleeding in moderate to severe cases. Diagnosis is based on clinical presentation and confirmed by genetic testing. Various local (nasal saline, air humidification, laser ablation, and electric cauterization for epistaxis and endoscopic Argon Plasma Coagulation-APC for active GI bleeding), surgical, and systemic (tranexamic acid and antiangiogenic agents like bevacizumab and thalidomide) treatment options are used depending upon disease severity. Here, we present a case with recurrent gastrointestinal bleeding refractory to endoscopic APC ablation and thalidomide and severe symptomatic anemia requiring multiple packed red cell transfusions. The patient was ultimately started on bevacizumab, to which he had a good response and has remained in remission for 8 months as of now. This case emphasizes the need to have a low threshold of suspicion to diagnose HHT and start targeted therapy like bevacizumab early on in moderate to severe cases of HHT rather than just relying on temporizing palliative measures like ablation, cauterization, and tranexamic acid.

## Introduction

Osler Weber Rendu Syndrome is an autosomal dominantly inherited disorder causing abnormal development of blood vessels leading to the formation of multiple telangiectasias and arteriovenous malformations (AVMs) involving mucocutaneous structures (lips, buccal mucosa, tongue, and fingertips) and viscera (lungs, liver, gut, and brain). Its prevalence ranges from 1:5,000 to 1:8,000, with an estimated 85,000 people affected in Europe (1, 2). Because of its autosomal dominant nature, it affects males and females fairly equally. However, some studies have concluded that females tend to develop more severe liver and pulmonary involvement than men (3). There are three main types of HHTs, which include Type 1, Type 2, and HHT-Juvenile polyposis overlap syndrome caused by mutations in ENG (endoglin), ACVRL1 (activin receptor-like kinase 1), and SMAD4 (transcription factor), respectively (4). These mutations affect TGFβ (transforming growth factor beta) pathway disrupting the key balance between angiogenesis inducers and inhibitors in favor of angiogenesis and consequent development of multiple telangiectasias and AVMs in various body regions.

Recurrent nosebleeds are common and occur in approximately 90% of affected individuals, mostly starting since childhood, while other symptoms include: gastrointestinal bleeding (25–30%) causing melena and severe symptomatic microcytic anemia; pulmonary AVMs (50%) causing dyspnea, hemoptysis, paradoxical emboli, and cerebral abscesses; cerebral AVMs (10%) causing headache, seizures, and focal neurological deficits; and hepatic AVMs (40–70%), which are mostly asymptomatic but can present symptoms of high output cardiac failure and hepatic decompensation with an ultimate requirement of liver transplantation (5). Although there is a wide array of manifestations ranging from asymptomatic to life-threatening, most of the time, the disease presents itself with a history of recurrent spontaneous epistaxis, symptomatic anemia (fatigue, palpitations, dizziness, etc.), and in some cases, chronic GI bleeding.

“Curacao criteria” is used to clinically diagnose the patients as HHT, which include recurrent spontaneous nosebleeds, mucocutaneous telangiectasias, visceral involvement, and first-degree family history of HHT. If ≥ 3 criteria are met, the diagnosis is definitive, while if 2 are met, then it will be suspected HHT, and if fewer than 2 criteria are present, then it will be labeled as unlikely HHT (6). In suspected cases, diagnosis can be confirmed by genetic testing for disease-causing mutations. In addition, genetic testing can also be done to screen the family members for the presence of mutations in HHT genes.

Treatment of the disease is multimodal, primarily focusing on limiting the bleeding from various sites like the nose and gastrointestinal tract, and correcting the anemia with iron supplementation and blood transfusion when necessary. Nasal ointments or gels, saline spray, air humidification, laser ablation, oral tranexamic acid for nose, GI bleeds, and in severe cases of acute GI bleed resection anastomosis of the diseased segment of gut or embolization of diseased vessel: all these measures help prevent or stop bleeding and do not target the actual cause of the disease (i.e., disrupted vessel integrity and aberrancy of vasculature). A recent approach for moderate to severe disease with blood transfusion dependence has promoted anti-VEGF (vascular endothelial growth factor) agents like bevacizumab, pazopanib, and thalidomide, decreasing the frequency of bleeding, need for transfusion, and improving the overall quality of life. However, these systemic therapies may cause adverse effects like thromboembolism, hypertension, peripheral neuropathy, etc. Hence benefit to risk assessment should be done before their initiation.

A single center non-randomized phase 2 clinical trial on 24 patients with HHT and severe hepatic malformations reported a significant decrease in the median cardiac index from 5.05 L/min/m^2^ (range, 4.1–6.2: pretreatment) to 4.1 L/min/m^2^ (range, 3.0–5.1, *P* < 0.001) after initiation of bevacizumab at 6 months follow-up. The mean duration of epistaxis, which was 221 min per month (range, 0–947, *P* = 0.008: pretreatment) reduced to 43 min per month (range, 0–310: post bevacizumab therapy at 6 months follow-up) (7). This preliminary study suggests the possible efficacy of bevacizumab in patients with advanced forms of HHT affecting multiple organ systems. Our case report presents a treatment-resistant case of HHT, which was subsequently successfully treated with bevacizumab, as evidenced by a significant reduction in the number of epistaxis, and GI bleeds; and stabilization of hemoglobin levels with no need for blood transfusions. This case adds to the growing evidence of the significant efficacy of bevacizumab in advanced forms of HHT, which is yet to be statistically evaluated by a randomized, placebo-controlled, and double-blinded clinical trial.

## Case presentation

**FIGURE 1:**
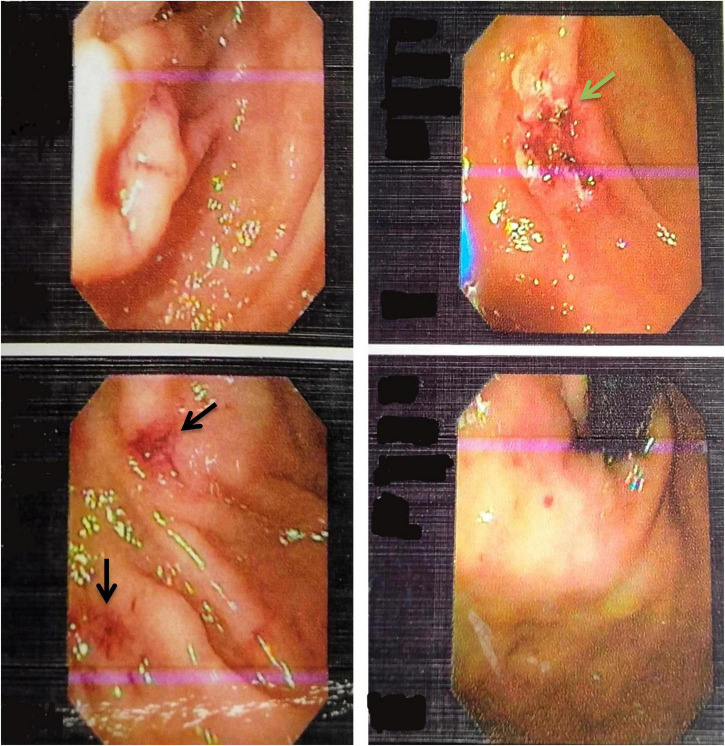
Upper Gastrointestinal endoscopy images showing multiple telangiectasia in the stomach (black arrows) and ulcer located in the second part of the duodenum (green arrow).

**FIGURE 2:**
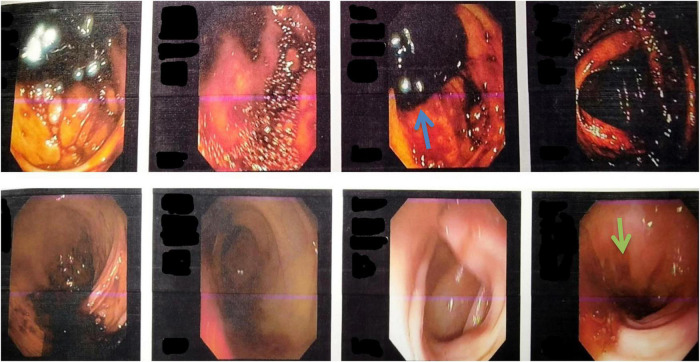
Colonoscopy images showing the presence of melanic stools in the cecum, ascending colon, and transverse colon (blue arrow), suggesting upper gastrointestinal bleed. The image also shows multiple telangiectasias in the rectosigmoid region (green arrow).

**FIGURE 3:**
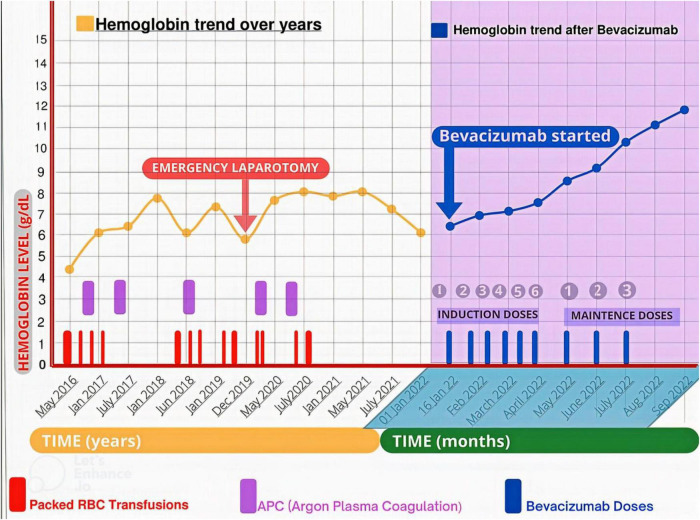
The trend in hemoglobin levels before and after bevacizumab initiation. The patient received multiple Packed RBC transfusions, and underwent multiple endoscopic argon plasma coagulation (APC) and an emergency laparotomy (for extensive GI bleed) to maintain hemoglobin level before bevacizumab commencement. While after initiating bevacizumab, he became transfusion independent and had a progressive uptrend in his hemoglobin level from 6.4 to 12 mg/dL at 8 months follow-up.

This 23-year-old patient with a positive family history of epistaxis in his (late) father had occasional episodes of epistaxis since childhood without seeking medical attention until adulthood (2016) when he presented with intermittent episodes of melena and severe generalized body weakness and palpitations. His lab investigations revealed positive fecal occult blood and very-low hemoglobin (Hb) of 4.6 g/dl requiring multiple transfusions with good clinical recovery. One year later (2017), he presented again with similar symptoms, for which an extensive work-up was done to find out the possible cause of iron deficiency anemia and thrombocytopenia. However, all work-up, including autoimmune profile, chromosomal study, stool microscopy, and bone marrow biopsy, were inconclusive of the cause showing only hypercellularity and megaloblastic changes with normal vitamin B12 and folate levels (Table 1). A gastrointestinal bleeding scan showed inconclusive findings of only tracer activity in the stomach and duodenum and further advised esophagogastroduodenoscopy (EGD). As per upper gastrointestinal endoscopy findings, positive fecal occult blood test, and normal colonoscopy, he was labeled as having gastritis and was given blood transfusions, iron supplements, and eradication therapy for *Helicobacter pylori*. In the following years, the patient had similar symptoms requiring multiple hospitalizations. In December 2019, he presented with gross per rectal bleeding. Evaluation with a CT angiogram showed hemangioma in one of the pelvic loops of the ileum. Excessive bleeding per rectum required urgent laparotomy, which revealed frank blood in the large and small gut loops and submucosal dilated vessels of about 2 cm in the mid ileum for which bowel loop resection was done 3 cm each proximally and distally with uneventful recovery. Histopathology of the specimen showed small dilated blood vessels with non-specific inflammation and was labeled as cavernous hemangioma based on these suggestive findings. His post-surgery abdominal CT-angiogram was normal. After 1 year, the patient presented again with per rectal bleeding and iron deficiency anemia along with thrombocytopenia, for which platelets and red cell transfusions were done with the initiation of steroids. He was refractory to steroids. The patient was further investigated in the gastroenterology unit with EGD showing multiple arteriovenous (AV) malformations in the gastroesophageal junction, gastric fundus, and pylorus and the second part of the duodenum with active bleeding and ulcerations for which argon plasma coagulation (APC) was done (Figure 1). In addition, a colonoscopy revealed AV malformations in the rectosigmoid region for which APC was done (Figure 2). The patient was continuously dropping his hemoglobin levels requiring multiple transfusions (Figure 3) and was refractory to multiple treatments (APC, steroids, and bowel resection of dilated vessels). After carefully reviewing the patient’s medical history and family history and the features of endoscopy by gastroenterologists and hematologists, a diagnosis of Osler Weber Rendu syndrome was made. The patient was subsequently started on bevacizumab and showed dramatic improvement (Figure 3) both symptomatically and hematologically, requiring no further transfusions. As a workup to look for any complications associated with Osler Weber Rendu syndrome, a CT angiogram of the brain and chest was done, which was normal. In addition, the patient’s virology and cytogenetic studies were normal. The patient has been closely followed since then and monitored with monthly CBC (complete blood count), as shown in Figure 3.

**TABLE 1:** Lab investigations done for the patient.

S. No	Entity of investigation		Results	Normal range
1	TLC		1.55 × 10^3^/uL	4–11 × 10^3^/uL
2	DLC	Neutrophils	52.3%	40–75%
		Lymphocytes	36.8%	20–45%
		Monocytes	7.7%	2–10%
3	Platelets		34 × 10^3^/uL	150–450 × 10^3^/uL
4	Hemoglobin		6.1 g/dL	11.5–17.5 g/dL
5	MCV		78.3 fL	76–96 fL
6	RDW%		17.2%	11.5–14.5%
7	Peripheral smear	Anisocytosis	+	
		Microcytosis	+	
		Hypochromic	+	
		Target cells	+	
		Giant platelets	+	
8	Coagulation profile	PT	16 s	12 s
		INR	1.3	1
		aPTT	28 s	28 s
9	Serum ferritin		74°ng/ml	30–400°ng/ml
10	Stool routine examination		Occult blood (+)	–
11	Iron studies	Iron	27 mcg/dL	60–70 mcg/dL
		TIBC	336 mcg/dL	240–450 mcg/dL
		Transferrin saturation	9.2%	20–50%
12	Bone marrow biopsy		Hypercellular marrow, M:E ratio = 2:1, Absent iron stain, Erythropoiesis-Hyperplastic with megaloblastic changes Diagnosis: Iron deficiency anemia with concurrent megaloblastic changes.	

TLC, Total Leukocyte Count; DLC, Differential Leukocyte Count; MCV, Mean Corpuscular Volume; RDW, Red cell Distribution Width; PT, Prothrombin Time; INR, International Normalized Ratio; aPTT, activated Partial Thromboplastin Time; TIBC, Total Iron Binding Capacity; M:E, Myeloid to Erythroid Ratio; uL, microliter.

### Treatment done and its response

When he was admitted to the hospital after severe anemia and thrombocytopenia, he received mega platelet infusion, red cell concentrate infusions, iv antibiotics, fluids, painkillers, and antipyretics for primary resuscitation and initial stabilization to initiate the bevacizumab therapy. The patient received the first injection of 300 mg bevacizumab (5 mg/kg body weight dose) during hospitalization, and he was subsequently discharged on home medications (Ferrous sulfate, folic acid, omeprazole, and antibiotics). During the induction phase of treatment, he received 300 mg of bevacizumab (5 mg/kg body weight) every 2 weeks for 2.5 months. His hemoglobin showed improvement from 6.4 mg/dL to 7.5 mg/dL. Seeing the dramatic improvement, he was subsequently put on maintenance treatment of 300 mg bevacizumab every 4 weeks for 3 more months (Table 2). His hemoglobin continued its uptrend and was 10.3 mg/dL at the 6-month follow-up visit (July 2022) after the initial presentation and maintenance therapy ended at this point. At 8 months follow-up (September 2022), his hemoglobin was 12 mg/dL (Figure 3). He was satisfied with the outcome of bevacizumab and reported a positive effect on his vitality and psychosocial wellbeing. The patient received nine doses of bevacizumab and had no significant side effects or major bleeding requiring blood transfusion.

**TABLE 2:** Dose and frequency of administration of bevacizumab and follow-up parameters.

Bevacizumab dose (mg/kg body wt.) (T_*x*_ phase)	Administration frequency	Number of doses	Hemoglobin (g/dL) (Start-End)	Number of PRBC trans.	Any bleeding episode	Average blood pressure (mm Hg)	Urine R/E for proteins
5 (induction)	Every 2 weeks	6	(6.4–7.5)	0	None	120/60	Negative
5 (maintenance)	Every 4 weeks	3	(7.5–10.3)	0	Mild epistaxis	130/80	Negative

T_x_, Treatment; PRBC trans, Packed Red Blood Cells transfusion; R/E, Routine Examination. The progressive rise in Hemoglobin levels (6.4–10.3) with transfusion independence after bevacizumab initiation.

## Discussion

Osler Weber Rendu syndrome is an autosomal dominant vascular disorder that usually presents with epistaxis, gastrointestinal bleeding, melena, iron deficiency anemia, pulmonary hypertension, telangiectasia, or arteriovenous malformation in the mucosa of GIT, liver, lungs, kidneys, and sometimes in the brain. It affects up to 1–20 cases/100,000, so always difficult to diagnose (8). HHT is a rare disease that is often misdiagnosed and mistreated before its diagnosis is established. A retrospective study from Italy reported a diagnostic delay of 25.7 years in the case of HHT, mainly due to the heterogeneous nature of the disease, lack of reliable biochemical tests, and awareness about the disease among the public (9). Therefore, it is essential to diagnose the disease early and manage it appropriately to prevent morbidity and mortality from visceral AVMs. The differential diagnosis includes bleeding disorders (von Willebrand disease, and hemophilia), vascular anomalies (angiodysplasia of the GI tract, mucocutaneous angiomas, CREST syndrome, and Kaposi sarcoma), and causes of lower GI bleeding (polyps, diverticulosis, and malignancy). Diagnosis of HHT is mainly clinical following the Curaçao Diagnostic Criteria, while genetic testing may be required in suspicious cases. Therefore, for proper diagnosis, one shall take a proper history of any bleeding episodes and symptoms of anemia along with family history and adequately examine the patient for any mucocutaneous telangiectasia. In case of GI bleeding, one shall do colonoscopy and endoscopy to look for AV malformations. Computed Tomography angiography and video-assisted capsule endoscopy can be used in complex cases of GI bleeding when colonoscopy and endoscopy findings are inconclusive to localize and determine AV malformations’ size and site of involvement. Anemia occurs due to the rupture of thin-walled distended venules under high pressure due to the absence of intervening capillaries leading to chronic iron deficiency anemia (10). Once diagnosed, there are different treatment modalities depending upon the severity of the disease. Systematic pharmacologic therapy options include antifibrinolytics (tranexamic acid), estrogen receptors modulators (tamoxifen and raloxifene), vascular endothelial growth factor inhibitors (bevacizumab, pazopanib, and sorafenib), immunomodulators (thalidomide, lenalidomide, and pomalidomide)—localized physical treatment include sclerotherapy, laser ablation, radiologic guided embolization, and surgical removal of AV malformations (11, 12).

In our case, the patient had epistaxis, GI bleeding, anemia, AV malformations in colonic and gastric mucosa, and a family history of epistaxis in his (late) father, fulfilling the Consensus criteria or Curacao criteria for diagnosis of HHT. The patient was refractory to multiple therapies like laser ablations, surgical resection of AVMs, steroids, and thalidomide 100 mg for 7 months. The patient was subsequently put on bevacizumab, considering his treatment-resistant course. The patient had a dramatic response to bevacizumab as evidenced by less frequent episodes of melena and epistaxis, almost no need for blood transfusion to maintain his hemoglobin levels, increase in hemoglobin levels from 6.4 to 12 mg/dL at 8 months follow up. A retrospective study by Iyer et al. concluded that bevacizumab effectively reduced the epistaxis severity score and need for blood transfusion in treatment-resistant HHT patients. New onset or worsening hypertension in four patients was a notable side effect of bevacizumab in their cohort of 34 patients (13). A recently published study by Vázquez et al. on HHT patients with severe hepatic involvement showed that bevacizumab could be helpful in HHT patients with High Output Cardiac Failure (HOCF) as a bridging therapy for liver transplantation by reducing the cardiac index and improving pulmonary hypertension (14). Most studies have used a 5–10 mg/kg body weight dose of bevacizumab (15–17). A prospective non-comparative study evaluated the efficacy of various bevacizumab maintenance regimens in controlling epistaxis duration and cardiac index. The results revealed that a monthly maintenance regimen with 5 mg/kg body weight was the most effective (18). On the other hand, some studies have proposed a much lower dose of bevacizumab (1 mg/kg/body weight every 2 weeks and 0.125 mg/kg body weight monthly) to reduce the number and duration of epistaxis (19, 20). Their dosing rationale is that dose-dependent side effects like hypertension and proteinuria may be avoided by lowering the dose, and these regimens may prove to be cost-effective. A comparative randomized placebo-controlled trial is needed to establish the optimum dosing schedule that achieves disease remission with minimal side effects and is also cost-effective.

It is essential to consider some of the side effects of these treatment modalities, as some may cause thromboembolic complications, hypertension, proteinuria, bowel perforation, infection, delayed wound healing, peripheral neuropathy, etc. It is recommended to regularly check blood pressure and urine for proteinuria in patients receiving bevacizumab. If a patient has to undergo surgery, then bevacizumab should be stopped 2 months before surgery and restarted 1 month after the surgery as it delays wound healing and increases the risk of infections (17). Hence, we should always consider the risk–benefit profile of these treatment modalities on an individual basis, and they should be used in the advanced treatment-resistant stage of HHT. The adult patient population suffering from HHT usually has an average or slightly reduced life expectancy. One study concluded a 3-year median reduction in age at death in patients suffering from HHT compared to the general population (21). Multidisciplinary involvement, including otorhinolaryngologists, gastroenterologists, interventional radiologists, hematologists, pulmonologists, and geneticists, is crucial to the early diagnosis and optimal management of HHT patients.

Our study adds to the growing evidence of the efficacy of bevacizumab in treatment-resistant cases of HHT. The patient dramatically responded to bevacizumab with almost no side effects and achieved blood transfusion independence. Diagnosis of HHT might be challenging in children and young adults where due to the evolution of the disease, all four clinical signs and symptoms of Curaçao criteria may not be apparent. Genetic testing might be useful to get an early diagnosis and improve long-term morbidity and mortality in such cases. Our study has some limitations like we did not have an adequate sample size, a placebo arm, and adequate follow-up time to compare the long-term objective measures of efficacy. A randomized, placebo-controlled, and double-blinded trial is warranted to establish the long-term clinical efficacy, optimum dosing schedule, and cost-effectiveness of bevacizumab compared to other treatment modalities.

## Conclusion

Early diagnosis of such rare diseases that require extensive work-up and low suspicion of diagnosis in the face of economic challenges of patients in developing countries is crucial. In challenging cases of GI bleeding, utilization of capsule endoscopy should be considered early, as our case had missed AV-malformation in one of the loops of the ileum, which could have been picked by capsule endoscopy. Additionally, for patients who require multiple endoscopies and colonoscopies, which are relatively discomforting, this modality of investigation should be taken into consideration. Early use of bevacizumab should be considered in moderate to severe diseases, and more studies should be done to outline its role and effectiveness in the treatment of HHT. GI ulcers not responding to the *H. pylori* eradication regimen should be looked at more carefully. Suspicion of having HHT should still be considered, especially in young individuals, if there are no obvious skin manifestations, as our case lacked the typical mucocutaneous telangiectasias.

## Data availability statement

The original contributions presented in this study are included in the article/supplementary material, further inquiries can be directed to the corresponding author.

## Ethics statement

Written informed consent was obtained from the individual(s) for the publication of any potentially identifiable images or data included in this article.

## Author contributions

HY, SA, and FH conceived the idea and conceptualized the study. HY wrote the Abstract and added tables and figures along with appropriate captions. SA contributed to writing the case presentation. FH assisted in writing the discussion section. WQA wrote the Introduction section. TH collected patient data and wrote the Conclusion section. WJA made a treatment chart and bevacizumab therapy response curve. BU did post manuscript editing and helped with arranging details of the case. PB contributed to drafting the revised manuscript on basis of the reviewer’s comments and removed grammatical mistakes from the final draft. All authors contributed to manuscript revision, read, and approved the submitted version.
